# Sustainable Food Production and Nutraceutical Applications from Qatar Desert *Chlorella* sp. (Chlorophyceae)

**DOI:** 10.3390/ani10081413

**Published:** 2020-08-13

**Authors:** Rihab Rasheed, Imen Saadaoui, Touria Bounnit, Maroua Cherif, Ghamza Al Ghazal, Hareb Al Jabri

**Affiliations:** Algal Technologies Program, Centre for Sustainable Development, Qatar University, P.O. Box 2713 Doha, Qatar; rihabrasheed@qu.edu.qa (R.R.); touria.bounnit@qu.edu.qa (T.B.); cherif.maroua@qu.edu.qa (M.C.); ali88@qu.edu.qa (G.A.G.); h.aljabri@qu.edu.qa (H.A.J.)

**Keywords:** biomolecules, food, *Chlorella*, proteins, PUFAs, thermotolerance

## Abstract

**Simple Summary:**

The world population is increasing rapidly, putting pressure on the existing resources, especially in the food sector. Besides having a continuous supply of food, it is also important to identify food sources that are nutritionally beneficial to the health of animals, fish, and humans. Hence, a balanced feed supplement including all the essential nutrients, such as amino acids, omega fatty acids, sugars, and carotenoids, is required. In this context, we have studied the beneficial effects of using a microalga, *Chlorella* sp. isolated from the desert environment in Qatar, as a potential feed supplement. We found that the local strain showed a rich biochemical profile, with high amounts of proteins and lipids having biomolecules essential for human. Furthermore, the hexane extracts of this strain showed high antiproliferative activity against leukemia, one of the deadliest cancers in the world, proving its potential in the path of drug discovery against cancers. Our research findings thus highlight the nutraceutical potential of the local *Chlorella* sp., which can resist high temperatures of up to 40 °C, making it suitable for a large-scale production in ponds, year-round, for use as a sustainable food supplement.

**Abstract:**

Microalgae isolated from the Qatari desert was identified as thermotolerant, with a rich metabolite profile that is appropriate for use as food and health supplements. In this research, a species of *Chlorella*, QUCCCM3, from the Qatar University Culture Collection of Cyanobacteria and Microalgae, was investigated for its growth characteristics and metabolite compositions for use as potential feedstock for food production. The strain was cultivated at 30, 35, and 40 °C, covering the annual average low and high temperatures in Qatar. The highest growth rates were recorded for cultures at 30 °C with 0.64 ± 0.04 day^−1^, followed by a growth rate of 0.54 ± 0.06 day^−1^ at 40 °C, indicating its thermotolerance ability. The biomass exhibited a high protein content (43 ± 2.3%), with existence of lysine (4.13%) as an essential amino acid, and docosahexaenoic acid, linoleic acid, and alpha-linolenic acid as important omega fatty acids present. On the other hand, *Chlorella* sp. QUCCCM3 also exhibited a high capacity for scavenging free radicals with an antiproliferative effect against chronic myeloid leukemia K562 cancer cells. The results indicate that *Chlorella* sp. QUCCCM3 is a promising candidate that can be produced year-round, in the Qatar environment, for commercial applications such as feed and nutraceutical supplements.

## 1. Introduction

Microalgae biomass is highlighted as a promising resource for different applications. Energy industries are exploring their potential as biodiesel, bioethanol, and biogas [1]. However, the economic feasibility of these processes remains questionable [2]. It is the nutrition sector that is ideally utilizing these organisms as potential food and feed sources.

Microalgae are mini factories, synthesizing important metabolites, such as proteins, carbohydrates lipids, essential amino acids, carotenoids, polyunsaturated fatty acids, and essential vitamins. These biological components are gaining importance as sustainable sources of feed, to satisfy the nutritional demands of a growing population [3,4,5]. The ever-increasing demand for animal proteins has put tremendous pressure on the existing resources of poultry, cattle, and fish. Alternately, the high protein content in microalgae has favored their usage as feed [6]. In addition, the ability of microalgae to produce all essential amino acids is an added highlight in the quality of these proteins. It has been proved earlier that the quality of such proteins is much higher than conventional plant, milk, and soy proteins [3]. Carbohydrates are also an important class of metabolites in microalgae. Carbohydrates constitute a large proportion of the diets mainly used to feed cattle and livestock. They are known to provide the energy required for the animals and the microbes in their rumen, which is necessary to maintain a healthy gastrointestinal tract. Microalgae also synthesize a wide range of essential fatty acids that have a positive impact on the health of animals. Being primary producers of these fatty acids emphasizes the importance of enriching the animal feed by using such microalgae. The most important polyunsaturated fatty acids (PUFAs) comprise docosahexaenoic acid (DHA, 22:6, n-3), eicosapentaenoic acid (EPA, 20:5, n-3), arachidonic acid (ARA, 20:4, n-6), and α-linolenic acids (ALA, 18:3, n-3). All of these fatty acids show multiple health benefits, uncluding in the treatment of cardiovascular disease and development of brain cells [7].

Microalgae are already known to support all the growth stages for marine and land animals [5]. When microalgae are added to the diets of domestic livestock, they confer numerous health benefits, such as improving immune response and disease resistance, promoting growth, enhancing milk production, increasing quality of meat and eggs, and antiviral and antibacterial activities [8]. Previous research work done on poultry highlighted that incorporating up to 15% algae in poultry rations is beneficial for their growth, with no adverse effects on their health. Eggs fortified with DHA via selective feeding of hens are already popular in markets [9,10,11,12]. Moreover, microalgae are commonly used in aquaculture, since they play a foundational role in the aquatic food chain, and they are easily ingested and assimilated by fishes and gastropods [13]. Fish fed with up to 10% algae exhibit enhanced growth and development [14]. Microalgal biomass rich in carbohydrates is an optimal source of energy for juvenile oysters and a few larval stages, including in *Patinopecten yessoensis* (Jay) scallop larvae [3,12].

Besides nutritional benefits, microalgae also have high potential for pharmaceutical applications. This is due to the antioxidant properties of PUFAs and another class of secondary metabolites, such as carotenoids, found in microalgae. Omega-3 fatty acids and carotenoids are known for their health benefits in cases related to cancer and cardiovascular diseases [15]. The combined effects of nutritional and pharmaceutical benefits of microalgae contribute to their nutraceutical aspect as feed.

The common species of algae used as food supplements include *Arthrospira* sp., *Dunaliella salina*, *Chlorella* sp., and *Tetraselmis* sp. [16]. Among them, freshwater strains, such as *Chlorella* sp., are known to produce high amounts of proteins and other molecules with antioxidant properties, such as omega-3 and omega-6 fatty acids [17,18]. Several studies have shown that *Chlorella* biomass has a positive influence on the animals when used as feed, due to its biochemical composition, palatability, and higher digestibility of nutrients. Similar positive impacts were recorded in aquaculture when the biomass was used as feed. These factors have favored their economical use as potential animal-feed constituents [19].

Qatar is a peninsula that is governed by a hot and humid climate for most of the year. Food security and self-sufficiency in food products is of prime importance in the country. In our current research, a *Chlorella* sp. QUCCCM3, isolated from the local desert environment, was evaluated for its thermotolerance capacity, biomass productivity, metabolites content, antioxidant capacity, and antiproliferative properties. The strain was thus characterized to determine its suitability as feed component, to improve the health and nutritional value of the animals.

## 2. Materials and Methods

### 2.1. Morphological Characterization Chlorella sp. QUCCCM3

*Chlorella* sp. QUCCCM3, a freshwater microalgae, isolated from Qatar’s Desert environment, belonging to the Qatar Culture Collection of Cyanobacteria and Microalgae [20] was selected for the present study. The morphology of *Chlorella* sp. QUCCCM3 was studied by using light microscopy (Primo star HAL microscope, full Kohler, Carl Zeiss, Germany), under 100× magnification. The strain was observed for its features, under two different abiotic stress, namely (i) continuous light and (ii) high temperature (40 °C). Culture at 30 °C and a photon flux density of 200 µmol photons m^−2^ s^−1^ with 12:12 dark: light cycles were used as control.

### 2.2. Microalgae Cultivation and Growth Analysis

*Chlorella* sp. QUCCCM3 was cultivated by using BG11 freshwater growth medium. The composition of the medium was as follows: NaNO_3_ (1500 mg L^−1^), K_2_HPO_4_·3H_2_O (40 mg L^−1^), MgSO_4_·7H_2_O (75 mg L^−1^), CaCl_2_·2H_2_O (36 mg L^−1^), Na_2_CO_3_ (20 mg L^−1^), citric acid (6 mg L^−1^), Ferric ammonium citrate (6 mg L^−1^), EDTA (1 mg L^−1^), H_3_BO_3_ (2.86 mg L^−1^), MnCl_2_·H_2_O (1.81 mg L^−1^), ZnSO_4_·7H_2_O (0.222 mg L^−1^), CuSO_4_·5H_2_O (0.079 mg L^−1^), Na_2_MoO_4_·2H_2_O (0.390 mg L^−1^), and Co(NO_3_)_2_·6H_2_O (0.049 mg L^−1^) [21,22]. All components were obtained from Sigma-Aldrich, for research use.

A single colony of *Chlorella* sp. QUCCCM3 was used to inoculate 5 mL of BG11 liquid media, which was incubated for 7 days at 30 °C, under an agitation of 200 rpm and a photon flux density of 200 µmol photons m^−2^ s^−1^, in an illuminated shaker (Innova^®^ 44R incubator shaker, New Brunswick Scientific, Ocala, FL, USA). The culture was gradually scaled up to a volume of 500 mL and incubated under the previously mentioned conditions of growth, with an initial optical density at 750 nm (OD_750nm_) of 0.2. A daily assessment of the OD_750nm_ was performed, using a spectrophotometer (Jenway 73100, Staffordshire, UK), for which 1 mL of the culture was taken and measured against a blank of only media without the cells. All cultures were done in duplicate, with two flasks, in the same experiment. The culture was harvested after 12 days of cultivation, at the onset of the stationary phase. The biomass was harvested and then freeze-dried prior to being subjected to assessment of its metabolite content. 

Dry weight was determined in replicates [23], for which 10 mL aliquots were collected and filtered through 0.47 mm cellulose nitrate membrane filter (Whatman, Darmstadt, Germany). Filters were dried at 80 °C for 24 h, and then transferred to desiccators over silica gel, for dehydration, until stable weight was recorded.

The growth rate (µ) was determined by using the following formula [24]:

$$µ=\frac{lnN_{2}-lnN_{1}}{T_{2}-T_{1}}$$

where *T*_1_ is the time corresponding to the beginning of the exponential phase; *T*_2_ is the time corresponding to the end of the exponential phase; *N*_1_ is OD_750nm_ at *T*_1_; and *N*_2_ is OD_750nm_ at *T*_2_.

Biomass productivities were calculated based on biomass dry weight, using the following formula [25]:

$$P=\left(X_{2}-X_{1}\right)/\left(T_{2}-T_{1}\right)$$

where *X*_2_ is biomass concentrations (g L^−1^) at T_end_ (end point of culture), and *X*_1_ is the biomass concentration (g L^−1^) at T_initial_ (start point of cultivation). All measurements were performed in triplicates.

### 2.3. Thermotolerance Study

For the study of the thermotolerance capacity, *Chlorella* sp. QUCCCM3 was scaled up gradually, as previously described (Section 2.2), to 1 L in bioreactors (DASGIP parallel bioreactor system, Eppendorf Inc., Ocala, FL, USA), with an initial OD_750nm_ of 0.2. The strain was subjected to four culture temperatures, nameley 30, 35, 40, and 45 °C, with a mixing set to 200 rpm, using a pitch blade impeller. The illumination was provided by 3 internal DASGIP LED sticks having 3 channel emission spectrum (channel A, 660,780 nm; channel B, 572,625,640 nm; channel C, 453 nm). Set points were 2.00, 1.244, and 2.00 μmol photons·s−1 for channels A, B, and C, respectively, fixed for a photon flux density of approximately 200 µmol photons m^−2^ s^−1^, which is considered to be optimum for the standard cultivation of microalgae, at 12 h:12 h dark:light cycles. A daily assessment of the OD_750nm_ was performed to be able to compare the growth performance of the strain under the different temperatures tested.

### 2.4. Metabolite Extraction and Estimation

The microalgal biomass collected after 12 days of cultivation, corresponding to the end of exponential phase of culture, was washed with deionized water and freeze-dried (Labconco, Freezone, Kansas city, MO, USA) prior to being subjected for metabolite analysis. For total protein, 25 mg of dried microalgae was hydrolyzed overnight at 60 °C, using 5 mL sodium hydroxide (NaOH 0.1 M) from Sigma-Aldrich (St. Louis, MO, USA) [26]. The total protein content was determined for the supernatant by using colorimetric assay, using Folin ciocalteau reagent [27].

Total lipids were extracted from freeze-dried algae biomass, using the method of Folch, with some modifications, where freeze-dried biomass was treated with sodium chloride solution (0.88%) and incubated at 4 °C, in an adequate volume of methanol. Post overnight incubation, double the volume of chloroform (Analytical grade; Sigma-Aldrich, St. Louis, MO, USA) was added to the mixture, and cells were disrupted by tissue lyzer (Qiagen, Hilden Germany). The mixture was centrifuged at 5000× *g* for 5 min, and supernatant was transferred into a pre-weighed tube. The methanol chloroform extraction step was repeated to ensure complete removal of lipids from the biomass. The organic phase was separated from the aqueous phase by adding 0.88% of NaCl and mixing of the solution. The organic phase was collected, dried, and weighed, and the lipid content was measured gravimetrically [28,29]. The lipid content was calculated by using the following equation [30].

$$Lipid\;content\;\left(\%\right)=Total\;lipids\frac{g}{Dry}biomass\;\left(g\right)\times 100$$

Carbohydrates were estimated using by phenol sulphuric acid reagent [31]. For the extraction, freeze-dried biomass was dispersed in glacial acetic acid and incubated at 85 °C for 20 min, to remove all chlorophyll that can interfere with the colorimetric assay. The colorless pellet obtained after treatment was hydrolyzed, using hydrochloric acid (HCl 4 M), at 90 °C for 2 h. Finally, the supernatant was neutralized with water and subjected to calorimetric assay, using phenol sulphuric acid.

### 2.5. Amino Acid Profiling

Amino acids were quantified by using pre-column derivatization with O-phtalaldehyde (OPA) and 9-fluorenylmethyl chloroformate (FMOC) after methods as described in Reference [32]. Pre-weighed algal biomass (approximately 2 mg) was hydrolyzed in 100 µL 6 N HCl at 120 °C for 24 h [33]. Analysis was performed in replicate, dried, and resuspended in 100 µL 0.1 N HCl. A reaction blank (no biomass) and a known protein standard (bovine serum albuminc; Sigma# 1076192) were performed. The HPLC system (Agilent 1260, Santa Clara, CA, USA) included a programmable autosampler for fully automated sample handling, derivatization, and sample injection (30 µL). Amino acid derivatives were separated by reverse-phase high-performance liquid chromatography (RP-HPLC), using a 5 µm Hypersil amino acid–octadecyl silane column (AA–ODS; 2.1 × 200 mm, Thermo Fisher Scientific, Waltham, MA, USA), using the solvent system and gradient described by Zheng et al. [34]. Amino acids derivatives were detected by using a variable wavelength UV detector (Agilent 1260, Santa Clara, CA, USA) and an in-line fluorescence detector (Agilent 1260, Santa Clara, CA, USA). The sample was quantified against a 5-point calibration curve from dilutions prepared from a standardized mixture of L-amino acids (Sigma Aldrich # P0834, St. Louis, MO, USA). Sixteen amino acids were reported; due to deamination, asparagine and glutamine were reported with aspartate and glutamate, respectively, as ASX and GLX. Note that tryptophan cannot be not determined by this method. The system operations and data analysis were performed on Chemstation. The assay was capable of detecting amino acid derivatives between 1 and 100 nmol.

### 2.6. Fatty Acid Methyl Ester (FAME) Profiling

FAMEs were extracted via a one-step transesterification method, where a known amount of freeze-dried biomass was treated, using sulfuric acid (95%; analytical grade from Sigma-Aldrich) and methanol solution (H_2_SO_4_:CH_3_OH = 1:10), and sonicated for 10 min. This step was followed by heat treatment at 80 °C for 2 h. Further, the mixture was transferred to a tube containing 1 mL of distilled water and 3 mL of hexane: chloroform (4:1; hexane and chloroform from Sigma-Aldrich, St. Louis, MO, USA) mixture and centrifuged. The top layer containing FAME fractions was filtered and analyzed, using GD-FID (Shimadzu 2010 plus, Kyoto, Japan) [29]. Then, 2 µL of sample was injected into gas chromatography set at 100–240 °C/5 min holding time. The sample was separated by using 100 m column with He as a carrier gas. FAMEs were identified based on retention time observed, using Supelco standards obtained from Sigma-Aldrich (37 Component FAME Mix: Cat # Number 200-838-9, St. Louis, MO, USA).

### 2.7. Extraction of Bioactive Molecules and Estimation of Carotenoids

The microalgae culture was cultivated at a scale of 500 mL, using BG11 growth media. The strain was cultured at 30 °C, representing the low average annual temperature in Qatar and optimum temperature for best microalgal growth. The mixing was set to 150 rpm, under a photon flux density of 200 µmol photons m^−2^ s^−1^, with continuous light, 24:00 (day and night cycle), using an illuminated shaker (Innova^®^ 44R incubator shaker, New Brunswick Scientific, Ocala, FL, USA). Here, the only stress affecting the biomass was continuous light. The optical density of the culture was monitored at 750 nm, and the culture was harvested at the late stationary phase. Algae secondary metabolites (carotenoids) were extracted via maceration, using hexane (analytical grade; Sigma-Aldrich, St. Louis, MO, USA) as an organic solvent [35]. For the extraction, 100 mg of freeze-dried biomass was dissolved into 10 mL of hexane. The mixture was subjected to a 5 min treatment at 30 hz, using the TissueLyser II (Qiagen, #85300, Valencia, CA, USA) for cell disruption. Then, the solvent fraction with the carotenoids was collected after centrifuge. The extraction was repeated on the same biomass, using fresh solvent, until the pellet became colorless. The total crude extract was subjected to spectral scan, at a range of wave lengths between 200 and 800 nm, using the Synergy H4 Hybrid Multi-Mode Microplate Reader (Bio-Tek, Winooski, VT, USA). The absorbance at 480 nm was recorded, and the concentration of carotenoids in the extract was calculated based on the following equation [36]:

$$Carotenoids\;\left(\mathrm{ug}\;{\mathrm{mL}}^{-1}\right)=Abs\;480\;\mathrm{nm}\times 4$$

Further, the extract was dried, weighed, and dissolved in 1% dimethylsulfoxide (DMSO), to a concentration of 100 mg mL^−1^. The crude extract was stored at 4 °C, in the dark, prior to being used for the investigation of its antioxidant and antiproliferative capacities.

### 2.8. TEAC Assay (Antioxidant Capacity)

The Trolox Equivalent Antioxidant Capacity (TEAC) assay was performed by using the Sigma-Aldrich TEAC Kit (CS0790, St. Louis, MO, USA), in which a ferryl myoglobin radical is formed from metmyoglobin and hydrogen peroxide, which oxidizes the 2,2′-azino-bis (3-ethylbenzthiazoline-6-sulfonic acid) (ABTS), to produce ABTS+, a cation that is green in color and determined spectrophotometrically at 405 nm, using Synergy H4 Hybrid Multi-Mode Microplate Reader (Bio-Tek, Winooski, VT, USA). A blank was prepared consisting of all the reagents except the algal extract to auto zero any absorbance caused by the reagents.The assay reflects the ABTS+ radical-scavenging capacity, in which a decrease in absorbance was observed due to the antioxidant molecules in the extract tested. All work was performed in the dark, at room temperature [37,38].

### 2.9. Cancer Cell Culture and Determination of the Antiproliferative Activity

Human chronic myeloid leukemia K562 cells from ATCC were cultured in RPMI 1640 medium supplemented with 10% heat-inactivated fetal bovine serum (FBS), 100 IU/mL penicillin, 100 μg mL^−1^ streptomycin, and 2 mM ^l−^glutamine. All the reagents were obtained from Sigma-Aldrich, St. Louis, MO, USA. Cultures were maintained in a CO_2_ incubator (Thermo Fisher Scientific, Waltham, MA, USA,) at 37 °C. Cell viability was tested by using trypan blue dye exclusion method. The effects of *Chlorella* sp. QUCCCM3 extracts on proliferation of leukemia cancer cell line K562 CML (Chronic Myelogenous Leukemia) were determined by using colorimetric micro-culture assay with the 3-(4,5-dimethyl-2-thiazolyl)-2,5-diphenyl-2H tetrazolium bromide (MTT) as end point [39]. Antiproliferative activity was tested for a range of algal-extract concentrations of (0, 5, 10, 25, 50, 100, 250, and 500 μg mL^−1^), for an incubation period of 24 h. An additional well was prepared as a blank, containing 1% DMSO, allowing a final concentration of 0.1%. Such a concentration was previously described as safe for the cells [40]. Cell viability was measured through optical density, using a synergy H4 hybrid Multi-Mode Microplate Reader (Bio-Tek # H4MLFPTAD, Winooski, VT, USA).

### 2.10. Statistical Analysis

Data were expressed as the mean of two independent parallel experiments. One-way ANOVA was used to assess the differences amongst the biomass productivities obtained after the different treatments and also between the inhibition % of the leukemia cancer cells from K562, using the different concentrations of the *Chlorella* sp. QUCCCM3 crude extract. The standard error of mean values was calculated at *p* < 0.05 level of significance.

## 3. Results

### 3.1. Growth Performance and Thermotolerance Capacity of Chlorella sp. QUCCCM3 Isolate

**Figure 1:**
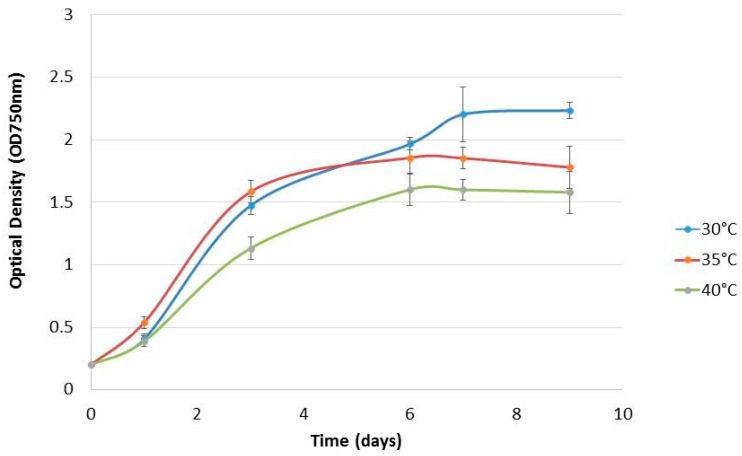
Growth curves of *Chlorella* sp. QUCCCM3 cultured under different temperatures. The points represent means ± SD of samples, *n* = 2.

*Chlorella* sp. QUCCCM3 was cultivated under different temperatures, namely 30, 35, 40, and 45 °C, knowing that 30 °C corresponds to the annual average temperature, and 40 °C to the high annual average temperature, in Qatar. *Chlorella* sp. QUCCCM3 presented a high growth rate of 0.64 ± 0.04 day^−1^ and a biomass productivity of 0.132 ± 0.01 g L^−1^ day^−1^, as shown in Table 1. Figure 1 showed that *Chlorella* sp. QUCCCM3’s growth decreased slightly with a temperature increase up to 40 °C; however, it exhibited identical values of ~0.54 °C day^−1^ at 35 and 40 °C. Similarly, the biomass productivity at 30 °C was significantly higher (*p* < 0.5) than those recorded for the cultivation at 35 and 40 °C, showing similar biomass productivities of 0.118 ± 0.02 g L^−1^ day^−1^ and 0.111 ± 0.01 g L^−1^ day^−1^, respectively (*p* > 0.05), whereas no growth was recorded for the strain when cultivated at 45 °C. Our results proved that *Chlorella* sp. QUCCCM3 is a fast-growing strain that can adapt at high temperatures, up to 40 °C, corresponding to the high average temperature in Qatar. This confirms its thermotolerance capacity and therefore its suitability to be cultivated year-round, at a large scale, under Qatar’s harsh climate.

### 3.2. Biochemical Composition of the Chlorella sp. QUCCCM3

**Figure 2:**
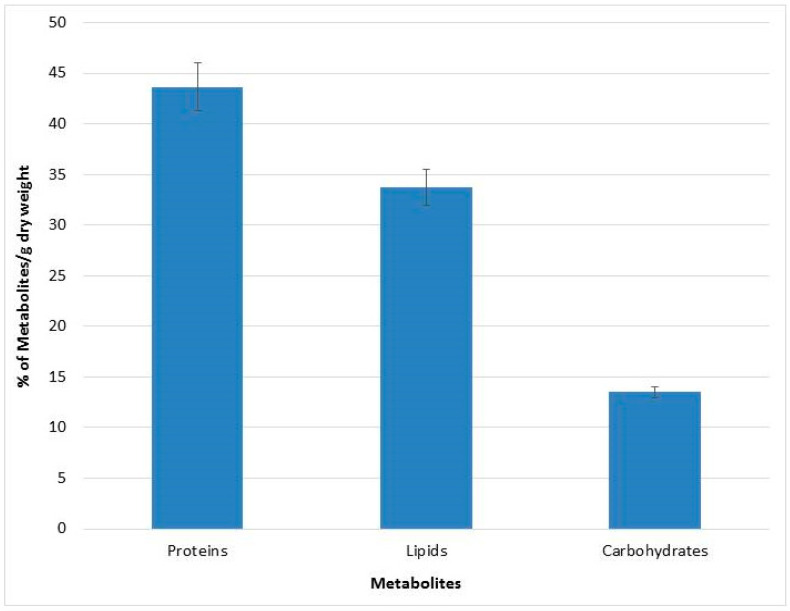
Metabolites characterization of *Chlorella* sp. QUCCCM3.

An assessment of the metabolites content was performed for QUCCCM3, to investigate its nutritional potential. The biomass obtained at 30 °C was subjected to quantitative and qualitative metabolite analysis. Results showed that *Chlorella* sp. QUCCCM3 presented a high amount of proteins, reaching up to 43 ± 2.3%, whereas the lipid amount was found to be 33 ± 1.75%. Additionally, the carbohydrates estimated was up to 13.5 ± 0.5%, as illustrated in Figure 2. Such results highlight the potential of our local isolate as a novel source of proteins and lipids.

**Figure 3:**
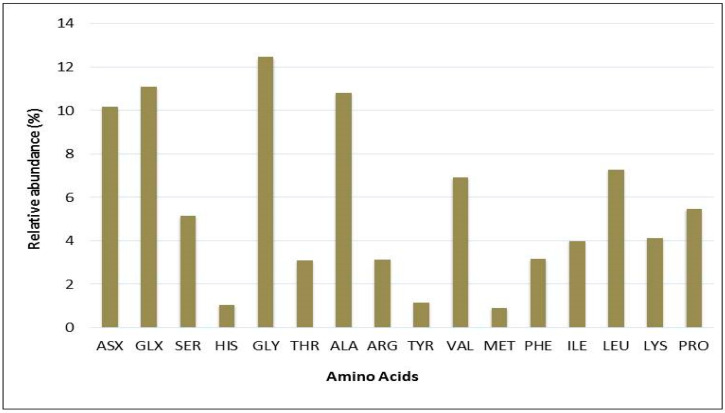
Percentages of total amino acid derivatives of *Chlorella* sp. QUCCCM3 protein lysate, obtained using the pre-column PITC labeling method, analyzed via HPLC-UV. ASX: Aspartic acid; GLX: Glutamic acid; SER: Serine; HIS: Histidine; GLY: Glycine; THR: Threonine; ALA: Alanine; ARG: Arginine; TYR: Tyrosine; VAL: Valine; MET: Methionine; PHE: Phenylalanine; ILE: Isoleucine; LEU: Leucine; LYS: Lysine; PRO: Proline.

To support these findings, the quality of proteins in *Chlorella* sp. QUCCCM3 was analyzed through amino acid profiling. The obtained data are illustrated in Figure 3 and confirmed the presence of the nine essential amino acids with different concentrations. Glycine was the most abundant essential amino acid, accounting for almost 14% of total protein. It also showed the presence of 12% GLX (representing both glutamate and glutamine, glutamate being precursor to L-glutamic acid). Leucine was present in concentrations up to 8%, and lysine, an important amino acid, was found to be 5% of the total proteins. The lowest concentrations of amino acids were found for histidine, tyrosine, tryptophan, and methionine. 

In addition to the amino acids, the FAME profile of *Chlorella* sp. QUCCCM3 was also determined, and the results are presented in Table 2. It was observed that the unsaturated fatty acids responsible for enhancing the nutritional value of the biomass were contributing up to 20% of the total fatty acid content, with the presence of essential fatty acids such as omega-3 and omega-6. The omega-3 fatty acids identified were DHA and ALA, and the omega-6 fatty acids included Linoleic Acid (LA). The principal FAME among the saturated fatty acids was palmitic acid, with a content of 167 ± 20.25 mg g dry weight^−1^ representing 35% of the total fatty acids. 

### 3.3. Characterization of the Strain Cultivated under Different Stress Regimes

**Figure 4:**
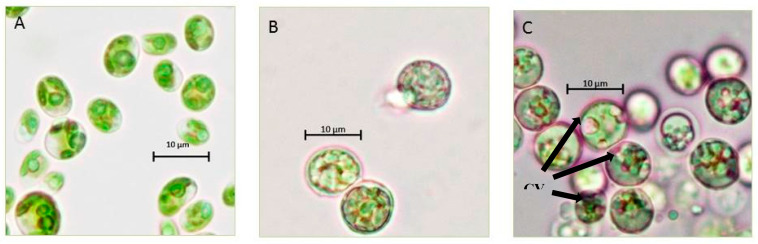
Morphological characterization of the *Chlorella* sp. QUCCCM3. Strains were cultivated under (**A**) control condition; (**B**) 40 °C; and (**C**) continuous light at 100× magnification, using light microscopy (Primo star HAL microscope, full Kohler, Carl Zeiss, Germany); CV: carotenoids vesicle.

*Chlorella* sp. QUCCCM3 showed a high growth rate and interesting biochemical profile, evidencing its suitability to be considered as a promising candidate for food supplement production. To further enhance its nutritional quality by increasing the amount of high-value added compounds associated with multiple health benefits, two different abiotic stressors, namely high temperature (40 °C) and continuous light, were applied separately. The comparative analysis of the cell morphologies when cultivated under both stresses, along with control condition, highlighted differences in the cell shape, size and, contents (Figure 4). 

The cells cultivated under both stresses showed a spherical shape instead of the normal oval shape. Furthermore, the cells cultivated under 40 °C presented a bigger size than the other previously cited conditions, whereas the microscopic observation of the cells cultivated under continuous light showed the presence of large orange vesicles. These vesicles correspond to the accumulation of carotenoids that are considered as high-value secondary metabolites. The existence of these carotenoids was confirmed by the presence of a peak of absorbance at 480 nm, in a spectral scan analysis. The concentration of this pigment was estimated to be 0.5 mg g^−1^ dry weight.

### 3.4. Antioxidant and Anticancer Potential of Chlorella sp. QUCCCM3

**Figure 5:**
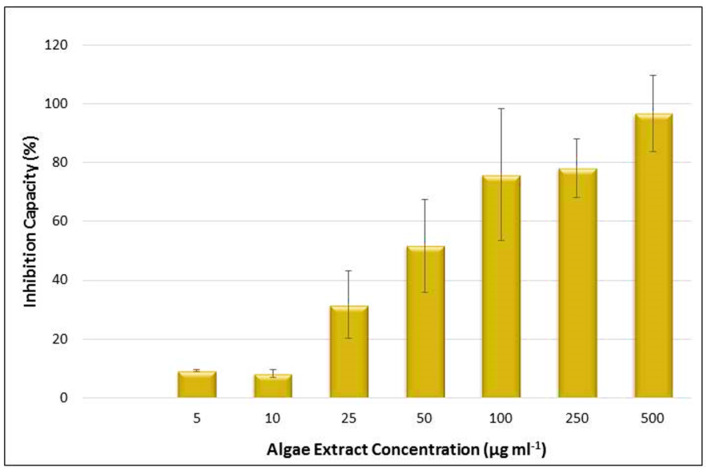
Inhibition capacity of the *Chlorella* sp. QUCCCM3 extract, using MTT assay against leukemia cancer cell line K562 after 24 h of incubation. Extracts used with concentrations of 5, 10, 25, 50, 100, 250, and 500 μg mL^−1^. Absorbance was measured at 540 nm, using the automated Microplate Spectrophotometer (Biotek). The blank-corrected absorbance of the control wells was taken as 100%, and the results were expressed as a percentage of the control. All data were recorded in triplicate, *n* = 3.

The presence of the essential fatty acids and carotenoids, which are already known for their nutraceutical properties and antioxidant activity, led to the investigation of the potential health benefits of the *Chlorella* sp. QUCCCM3. Hence, antioxidant and anticancer activities of the cell extract were assessed. Results showed interesting antioxidant activity with 65.2 ± 2.5 μmol g^−1^. In addition, the algal extract presented a high antiproliferative effect on the leukemia cancer cells K562, in a dose-dependent manner, as detected by MTT assay (*p* < 0.5). The hexane extract of *Chlorella* sp. QUCCCM3 showed an IC50 value of 21.37 ± 2.98 μg mL^−1^ (Figure 5). Such interesting findings evidenced additional health benefits of our local microalgae strain and proved its suitability for anti-leukemia drug discovery.

## 4. Discussion

The growth rate obtained for *Chlorella* sp. QUCCCM3 isolated from the desert was similar to the growth compared to *Chlorella* sp. isolated from different environments, such as springs and mountains, at a temperature of 30 °C, exhibiting a value of 0.66 day^−1^ [24]. However, the local *Chlorella* sp. QUCCCM3 exhibited its thermotolerance capacity, being able to grow at 40 °C, whereas the other *Chlorella* spp. were unable to survive even at 38 °C [37]. It is known that temperature is one of the parameter influencing the algae growth, and previous data highlighted a correlation between increase in temperature and growth of microalgae, such that growth is optimum at 30 °C [24,41]. It was seen that majority of the work on *Chlorella* sp. has been limited up to 35 °C. According to a few researchers [42], the optimum temperature for *Chlorella* sp. is 30 °C, and at 35 °C, the growth is declining, whereas in our research, we found that, although the growth slightly decreased at 35 °C (from 0.64 to 0.54 day^−1^), *Chlorella* sp. QUCCCM3 was stable up to 40 °C, with negligible variations in growth. Therefore, the strain is considered thermotolerant, as it can grow in higher temperatures, as compared to other known *Chlorella* strains. Its tolerance can be attributed to its origin of isolation and its evolutionary adaptation to a desert climate over the years, making it unique. Our findings also showed best biomass productivity of 0.132 ± 0.01 g L^−1^ at 30 °C for *Chlorella* sp. QUCCCM3, which was higher than the productivity recorded for other *Chlorella* spp. (0.114 ± 0.850 g L^−1^ day^−1^), as reported by different researchers [43]. It should be noted that the growth characteristics of this strain can vary when another variable such as light intensity is tested along with temperature. Several researchers have proved that there exists a correlation between the effect of temperature and light on biomass production, such that maximum growth can be achieved under optimum light and temperature interactions [44,45].

Microalgae are an important source for feed supplements and bioactives [46,47]. However, a key limiting factor in valorizing the microalgal biomass is its low productivity, which then reduces the economic feasibility of the biomass production for use as feed and nutraceuticals [48]. By presenting a high biomass productivity under normal growth conditions, *Chlorella* sp. QUCCCM3, demonstrated its capacity for high growth and biomass production under favorable cultivation conditions, thereby proving its commercial suitability. *Chlorella* sp. QUCCCM3 further exhibited a biomass concentration value of 1.72 ± 0.01 g L^−1^, which slightly decreased at 35 and 40 °C, although the cells still expressed normal growth with the rise in temperature, which is remarkable. A similar trend was confirmed by several other studies on *Chlorella* sp. [49,50].

In addition to the biomass productivity, metabolite production is the other imperative factor toward commercial utilization of microalgae for nutritional purposes [51]. The protein content found for *Chlorella* sp. QUCCCM3, 43 ± 2.3%, was higher than commercially available *Chlorella* sp., having a value of 39.98% [52]. Protein quality is determined by the presence of essential amino acids. These amino acids greatly increase the value of the biomass, since animals cannot synthesize such amino acids, and their nutritional requirements for these molecules are met through their feed. *Chlorella* sp. QUCCCM3 was enriched with all the essential amino acids with the existence of Lysine up to 5%, an important amino acid, indicating its suitability as potential feed. This amino acid also imparts a sweet flavor to the biomass, to improve the palatability for livestock, which is an important criterion for feed supplement production [53]. Similarly, in a different research study, it was proved that glutamate also contributes to flavor in the biomass. *Chlorella* sp. QUCCCM3 exhibited high amounts of glutamate, thus highlighting the sensory characteristics of the microalgae and improving the taste, when used as food ingredient [54].

In regard to the total lipids, the values reported for all *Chlorella* spp. ranged from 28 to 32% [55]. *Chlorella* sp. QUCCCM3 exhibited a slightly higher amount of lipids when compared to the given range of values. Precisely, the lipid content in *Chlorella* sp. QUCCCM3 was found to be much higher when compared to the amounts observed for *Chlorella pyrenoidosa* and *Chlorella vulgaris* which presented values around 2% and 14–22%, respectively [3]. The high amount of lipids was complemented by the presence of PUFAs, a key indicator for the nutritional potential of the strain. These long-chain fatty acids are known for their positive health benefits for animals and humans, including as antioxidants, anti-inflammatories, and anti-bacterial agents. The presence of DHA in the FAME profiling of *Chlorella* sp. QUCCCM3 biomass proved its commercial potential for producing feed for aquaculture [56]. It was stated that a microalgal diet enriched with DHA led to high growth rates of bivalve larvae, and its incorporation into poultry feed led to the production of omega-3-rich eggs [57,58]. Besides egg quality, dietary supplementation with 1–2% of DHA-rich microalgae has shown to have a positive effect on serum composition, caracass trait, antioxidant status, and fatty acid deposition in broilers [59]. Furthermore, the other omega-3 fatty acid found, ALA, is primarily seen in products such as vegetable oils and cereals. It is known that ALA, when consumed, gets assimilated in the body, to form EPA, which is a very important omega-3 fatty acid with multiple health benefits. *Chlorella* sp. QUCCCM3 also possesses LA, which is observed in high amounts in corn oil, sunflower oil, walnuts, brazil nuts, and soya beans, and hence can be used to replace the oils and provide the same nutritional benefits, without causing the side effects, such as cardiovascular diseases. Palmitic acid found among the FAMEs is known to improve the quality of milk by regulating milk fats [9,12]. In this context, we can anticipate that *Chlorella* sp. QUCCCM3 has the potential to be used as feed for livestock production. A similar trend was seen for Rhodophytas, which also exhibited high palmitic acid content. However, the amount was lower than the values found in microalgae [60]. The *Chlorella* sp. QUCCCM3 isolate from Qatar showed slight variations in its FAME profile, as compared to strains found in Australia [52]. While the presence of the omega-3 fatty acid, ALA, was observed in microalgae isolated from both environments, DHA was only found in *Chlorella* sp. QUCCCM3. Our local microalgae strain exhibited an omega-6/omega-3 ratio of 0.42, which is within the recommended range (0.25–1) for this strain to be beneficial also for human use [52]. The presence of important PUFAs (DHA and LA) showed that *Chlorella* sp. QUCCCM3 exhibits the cellular mechanisms for producing these compounds and thus can be modulated through variation of the cultivation regime [51,61].

With regard to carbohydrate content of *Chlorella* sp. QUCCCM3, it was within the range that is commonly seen for microalgae [62].

*Chlorella* sp. QUCCCM3 was subjected to abiotic stress, to enhance its nutraceutical potential. Morphological differences, such as changes in cell size and shape, were observed under both stresses applied separately. The interesting observation that was made about the cell size at 40 °C can be explained by a phenomenon called gigantism that was previously described for *Chlorella* sp. under temperature stress [63]. Moreover, it is suggested that increase of the cell size occurs to regulate the internal temperature required for maintaining normal cell functionality at higher temperatures. Our assumption is that this feature can allow the exchange of fluids between cells by increasing membrane porosity, to maintain the ambient temperature required by the cells.

*Chlorella* sp. QUCCCM3 microalgae also exhibited large circular vesicles of carotenoids under continuous light stress, as seen in Figure 4. Such structures were not visible for *Chlorella* sp. QUCCCM3 when cultivated under normal conditions (control) and at 40 °C. It has been proved widely that light is a key factor influencing the production of carotenoids within the cells. The accumulation of these pigments can be attributed to photo-induced activation of gene-encoding enzymes involved in the β-carotene biosynthesis [64]. The presence of these high-value secondary metabolites indicated their possible high antioxidant potential. The antioxidant value for the extract of *Chlorella* sp. QUCCCM3 was found to be higher than the value reported in other research using different *Chlorella* sp., exhibiting 65.2 ± 2.5 μmol g^−1^ and 56.09 ± 1.99 μmol g^−1^, respectively [65], although both extractions were made in different solvents, with the former being hexane and the latter being water. In a different study, hexane extract for our strain exhibited very high antioxidant potential when compared to hexane fractions for a large number of *Chlorella* spp., as reported for *Chlorella protothecoides* (3.49 ± 0.13 μmol g^−1^), *Chlorella pyrenoidosa* (8.48 ± 0.42 μmol g^−1^), *Chlorella vulgaris* (5.53 ± 0.05 μmol g^−1^), and *Chlorella zofingiensis* (1.83 ± 0.21 μmol g^−1^) [66]. The pigments, coupled with important fatty acids such as DHA, found in our strain are a prerequisite for characterizing *Chlorella* sp. QUCCCM3 as a nutraceutical supplement. The health benefits of these molecules are well-known, specifically as anticancer molecules [67]. For this reason, the antiproliferative activity of *Chlorella* sp. QUCCCM3 crude extract was also carried out, and the results showed an interesting IC50 below the standard limit described by the American National Cancer Institute (IC50 ≤ 100 μg mL^−1^). It has been recommended that lower the IC50 value, the higher the potential of the molecules for drug development is. Furthermore, this activity is about half of that described previously for *C. ellipsoidea* and *C. vulgaris* extracts, showing IC50 of 40.73 ± 3.71 and 40.31 ± 4.43 μg mL^−1^, respectively [68]. This interesting antiproliferative activity can be explained by the fact that *Chlorella* sp. QUCCCM3 is naturally adapted to produce secondary metabolites, such as carotenoids, to protect the cells when exposed to abiotic stressors common throughout the harsh Qatari climate. This also converges with other findings proving that microalgae, when under stress, developed components for survival and defence unique to them, in comparison to the terrestrial plants [69]. *Chlorella* sp. QUCCCM3 crude extract showed a peak of absorption at 480 nm, indicating the presence of carotenoids [70].

## 5. Conclusions

*Chlorella* sp. QUCCCM3 demonstrated unique characteristics and can be considered for year-around cultivation due to its thermotolerance capacity. In addition, the presence of high protein content with essential amino acids with high amounts of glutamate and lysine confirmed its nutritional potential. PUFAs, such as DHA and carotenoids, found in the local strain serve as powerful antioxidants. Furthermore, the extracts from the strain also have significant anticancer activity against Leukemia cancer cells, making them interesting from a pharmaceutical perspective. Therefore, *Chlorella* sp. QUCCCM3 can be considered to be a promising alternative that has potential for sustainable food production in an arid environment. 

## Figures and Tables

**Table 1:** Effect of temperature on growth parameters of the strain *Chlorella* sp. QUCCCM3. Number of replicates generating the mean, *n* = 2.

Culture Condition	Growth Rate (day^−1^)	Final Biomass (g L^−1^)	Biomass Productivity (g L^−1^ day^−1^)
30 °C	0.64 ± 0.04	1.72 ± 0.01	0.132 ± 0.01
35 °C	0.54 ± 0.06	1.06 ± 0.04	0.118 ± 0.02
40 °C	0.54 ± 0.01	1.00 ± 0.02	0.111 ± 0.01

**Table 2:** Fatty acid composition of *Chlorella* sp. QUCCCM3, a *C.* sp. isolate from Qatar, after cultivation in BG11 medium for 12 days at 30 °C. Profile obtained via GC–FID, using a trans-esterified sample.

FAME	FAME per g Dry Weight (mg g^−1^)
Myristic acid C14:0	4.01 ± 0.25
Palmitic acid C16:0	167 ± 20.25
Stearic acid C18:0	7.8 ± 3.7
Elaidic acid C18:1 n9t	16.17 ± 1.5
Oleic acid C18:1 n9c	38.19 ± 19.64
Linoleic acid C18:2 n6c	17.11 ± 15.2
Arachidic acid C20:0	94.56 ± 93.5
y-Linolenic acid C18:3n6	0.62 ± 0.3
Cis-11-Ecosenoic acid C20:1n9	4.29 ± 3.82
Cis-11,14-Eicosadienoic acid C20:2	2.03 ± 0.91
Behenic acid C22:0	2.38 ± 0.302
Lignoceric acid C24:0	0.74 ± 0.03
Docosahexaenoic acid C22:6n3	6.64 ± 0.377
